# Overexpression of *GbF3′5′H1* Provides a Potential to Improve the Content of Epicatechin and Gallocatechin

**DOI:** 10.3390/molecules25204836

**Published:** 2020-10-20

**Authors:** Yaqiong Wu, Tongli Wang, Yue Xin, Guibin Wang, Li-An Xu

**Affiliations:** 1 Co-Innovation Center for the Sustainable Forestry in Southern China, College of Forestry, Nanjing Forestry University, 159 Longpan Road, Nanjing 210037, China; yqwu@njfu.edu.cn (Y.W.); xiny@njfu.edu.cn (Y.X.); gbwang@njfu.edu.cn (G.W.); 2 Research Center for Pomology, Institute of Botany, Jiangsu Province and Chinese Academy of Sciences, Qian Hu Hou Cun No.1, Nanjing 210014, China; 3 Department of Forest and Conservation Sciences, Faculty of Forestry, University of British Columbia, Vancouver, BC V6T 1Z4, Canada; tongli.wang@ubc.ca

**Keywords:** F3′5′H, overexpression, flavonoid, metabolite

## Abstract

The flavonoids in *Ginkgo biloba* L. (*ginkgo*) have important medicinal uses due to their antioxidant, antitumor, and blood circulation-promoting effects. However, the genetic mechanisms underlying flavonoid biosynthesis in *ginkgo* remain elusive. Flavonoid 3′, 5′-hydroxylase (F3′5′H) is an important enzyme in flavonoid synthesis. We detected a novel differentially expressed *GbF3′5′H1* gene homologous to the F3′5′H enzyme involved in the flavonoid synthesis pathway through transcriptome sequencing. In this study, we characterized this gene, performed an expression analysis, and heterologously overexpressed GbF3′5′H1 in *Populus*. Our results showed that *GbF3′5′H1* is abundant in the leaf and highly expressed during April. We also found four metabolites closely related to flavonoid biosynthesis. Importantly, the contents of 4′,5-dihydroxy-7-glucosyloxyflavanone, epicatechin, and gallocatechin were significantly higher in transgenic plants than in nontransgenic plants. Our findings revealed that the *GbF3′5′H1* gene functions in the biosynthesis of flavonoid-related metabolites, suggesting that GbF3′5′H1 represents a prime candidate for future studies (e.g., gene-editing) aiming to optimize *ginkgo* flavonoid production, especially that of flavan-3-ols.

## 1. Introduction

Flavonoids represent a large class of secondary metabolites in plants and have many physiological functions [1]. Flavonoids are considered an important part of the plant chemical defense mechanism [2]. Flavonoid scaffolds are formed from the building blocks of a phenylpropanoid precursor (p-coumaroyl-CoA) and polyketide condensing unit (malonyl-CoA) by a series of reactions, including oxidation, reduction, condensation, and isomerization [3,4]. The scaffolds are then further modified to produce various subclasses of flavonoids by different classes of enzymes [5]. Some genes involved in flavonoid synthesis have been studied [6,7,8], and the biosynthetic pathway of flavonoids in Arabidopsis thaliana has been well-studied [9]. The Arabidopsis enzymes of the central flavonoid pathway are encoded by single genes, with the exception of flavonol synthase [4]. In contrast, the flavonoid biosynthetic pathway in *ginkgo* is complex, and each step is regulated by a multigene family. Recent evidence has revealed that flavonoid biosynthesis is regulated by several crucial enzyme-encoding genes [8]. Although the catalytic activities of several *ginkgo* homologs (GbCHS, GbCHI, GbF3H, and GbFLS) have been characterized in *Escherichia coli* [7,10,11,12], it is still unclear how these enzymes participate in flavonoid synthesis in plants because of the absence of related mutants.

*Ginkgo biloba* L. (*ginkgo*) is an ancient relic plant. As it is the only extant species in the division Ginkgophyta, it is considered to be a “living fossil” [13]. *Ginkgo* originated more than 200 million years ago, and it is a unique species on its own branch of the plant phylogenetic tree [14]. Due to its high environmental adaptability, stress tolerance, ornamental value, and medicinal utilization value, it has been introduced to many countries and regions [15]. Additionally, it is an important medicinal tree, because its leaves contain flavonoids and terpene lactones with useful pharmacological activities [16,17,18]. Currently, *Ginkgo biloba* extract (EGB) is one of the most famous products of this species and has been widely used for purposes, including the prevention of Alzheimer’s disease and cerebrovascular dysfunction disorders [19,20,21,22]. While there are many difficulties in the supply of ginkgo leaves, and chemical synthesis is far from meeting business production needs, there is great potential for development and economic value. Moreover, breeding to improve flavonoid productivity cannot be accomplished through genetic transformation, because no regeneration system has been established for *ginkgo* [23].

To better understand the process and mechanism of flavonoid biosynthesis, the transcriptome profiling of gingko leaves with different flavonoid contents was performed, and 457 genes were found to be significantly differentially expressed between a relatively high and a relatively low flavonoid content library (from the National Center for Biotechnology Information (NCBI) Short Reads Archive (SRA) database, accession number SRP137637) [18]. The flavonoid 3′,5′-hydroxylase (F3′5′H) enzyme gene has a wide range of flavonoid substrate activities and is involved in the biosynthetic pathways leading to flavonols (myricetin and quercetin) and flavan-3-ols (e.g., catechin, epicatechin, and epigallocatechin) [24,25]. Therefore, based on the annotated transcriptome sequences, we chose the differentially expressed *F3′5′H1* gene to further explore its important role in *ginkgo* flavonoid biosynthesis and metabolism. The *F3′5′H* gene, which encodes a cytochrome P450 monooxygenase (CYP) and belongs to the CYP75A subfamily, has been thoroughly investigated in the ornamental plant *Petunia hybrida* [6]. F3′5′Hs have also been isolated and characterized in various other plants, such as *Solanum lycopersicum* [3], *Vitis vinifera* [26], *Antirrhinum kelloggii* [27], and *Pericallis* × *hybrida* [28]. However, no reports of the cloning, characterization, and function of F3′5′H from *ginkgo* are available.

In this study, our major objectives were to characterize and functionally analyze the *F3′5′H1* gene of *ginkgo*, including molecular cloning, multiple alignment, phylogenetic analysis, expression patterns, and heterologous overexpression. In addition, a metabolomic analysis was performed on nontransgenic (CK) and transgenic seedlings to determine whether differentially expressed metabolites were linked to flavonoid biosynthesis and metabolism. The findings of this study not only provided information for the characterization and function of GbF3′5′H1 in this species but, also, helped increase the understanding of the underlying molecular mechanisms of flavonoid synthesis.

## 2. Results

### 2.1. Isolation and Characterization of Ginkgo GbF3′5′H1

**Figure 1:**
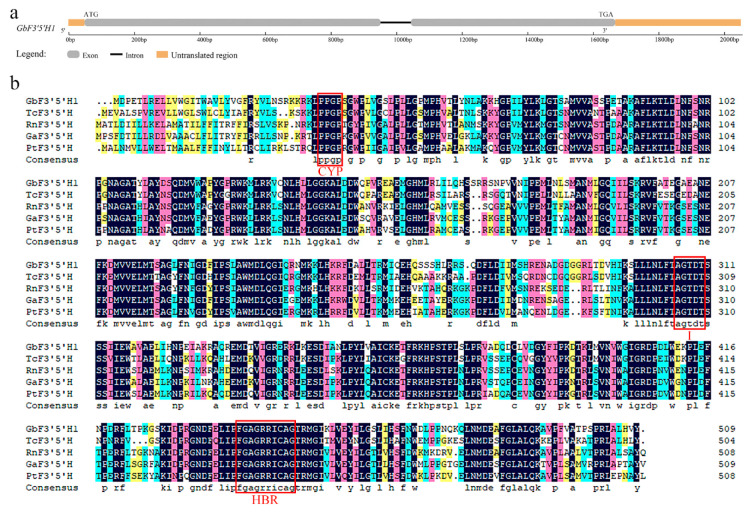
Sequence analysis of the *GbF3′5′H1* gene. (**a**) Structural characteristics of the *GbF3′5′H1* gene. The gray boxes represent the exons, and the thick black line represents the intron. (**b**) Alignment of the deduced amino acid sequence of GbF3′5′H1 and other known F3′5′Hs. The highly conserved regions of the F3′5′H proteins are shown by the three red boxes, which represent the following conserved domains: cytochrome P450 monooxygenase (CYP), the I helical region (I), and the heme-binding region (HBR). GbF3′5′H1, *Ginkgo biloba*; TcF3′5′H, *Taxus chinensis* (identity: 74.6%, GenBank: ATG29931.1); RnF3′5′H, *Ribes nigrum* (identity: 66.27%, GenBank: AGI16385.1); GaF3′5′H, *Gossypium arboreum* (identity: 67.60%, GenBank: KHF97502.1); and PtF3′5′H, *Populus trichocarpa* (identity: 64.97%, GenBank: XP_002314004.2). The dark blue color represents 100% identity. The red color represents 75% ≤ identity < 100%. The light blue color represents 50% ≤ identity < 75%. The yellow color represents 33% ≤ identity < 50%.

Based on annotated transcriptome sequences [18], we chose to further explore the important role of the differentially expressed F3′5′H protein in the flavonoid biosynthetic pathway and metabolism of *ginkgo*. To isolate the *F3′5′H* gene, 5′ RACE and 3′ RACE were performed to obtain the full-length cDNA for the putative *F3′5′H* gene, and the resulting gene was named *GbF3′5′H1* (accession number MN862535 in GenBank). The full-length sequence of this cDNA was 1959 bp, containing an open reading frame (ORF) of 1527 bp, flanked by a 42-bp 5′-untranslated region (UTR) and a 387-bp 3′-UTR; the termination codon was TGA (Supplementary Data 1). A comparison of the exon/intron structures of the *ginkgo* genomic DNA and cDNA sequences indicated that this gene contains two exons and one intron (no coding amino acid) (Figure 1a).

Bioinformatics software was used to predict the protein structure of *GbF3′5′H1*, and the results showed that the cDNA encoded a polypeptide comprising 509 amino acids. The molecular weight of the protein encoded by *GbF3′5′H1* was 57.33 kDa, and the theoretical isoelectric point (pI) was 9.19. The grand average of hydropathicity (GRAVY) was -0.185 (hydrophilic protein), and the aliphatic index was 94.85. The instability index (II) was computed to be 40.07, which classifies the protein as unstable. The TMHMM and Phobius software prediction showed that *GbF3′5′H1* had one transmembrane signal. Moreover, the SOPMA program was used to predict the secondary structure of GbF3′5′H1. The results indicated that the GbF3′5′H1 protein contained 48.92% alpha helices, of which 35.36% were random coils, 10.61% were extended strands, and 5.11% were beta turns. The InterPro online results classified the functional protein encoded by GbF3′5′H1 as a member of the CYP, E-class, group I (IPR002401) protein family.

### 2.2. Multiple Alignment and Phylogenetic Analysis of GbF3′5′H1

The multiple alignments of GbF3′5′H1 with F3′5′Hs from other plants showed that GbF3′5′H1 has many highly conserved residues compared with the known F3′5′Hs sequences (Figure 1b). This result revealed that F3′5′Hs contain the following three highly conserved regions: (i) the proline/proline/glycine/proline (PPGP) motif in the N-terminus, (ii) the I helical region “AGTDT”, and, (iii) the heme-binding region (HBR) near the carboxy (C)-terminus “FGAGRRICAG”. In addition, the multiple alignment analysis showed that the main difference between the amino acid sequence of GbF3′5′H1 and those of F3′5′H proteins in other species is the presence of approximately 30 amino acids at the N terminus, and this sequence mainly contains hydrophobic amino acid residues.

**Figure 2:**
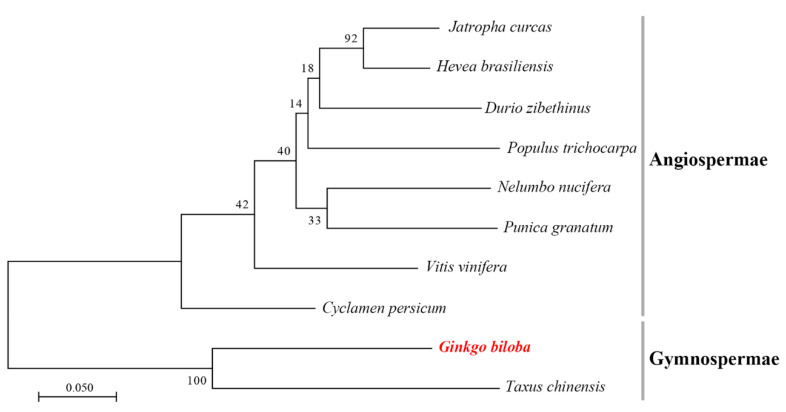
Phylogenetic tree of *F3′5′H* proteins from different species. The maximum likelihood method was used to construct the tree and was supported by bootstrapping based on 1000 replications. The following are the protein sequences used in these trees: *Hevea brasiliensis* (GenBank: XP_021664632.1), *Jatropha curcas* (GenBank: XP_012065863.1), *Punica granatum* (GenBank: AUY62559.1), *Durio zibethinus* (GenBank: XP_022756700.1), *Populus trichocarpa* (GenBank: XP_002314004.2), *Nelumbo nucifera* (GenBank: ARQ79447.1), *Vitis vinifera* (GenBank: BAE47007.1), *Cyclamen persicum* (GenBank: ACX37698.1), and *Taxus chinensis* (GenBank: ATG29931.1).

To understand the evolutionary relationships between the GbF3′5′H1 protein and the F3′5′H proteins of other species, the amino acid sequences of 10 F3′5′H proteins were aligned. A phylogenetic tree based on this multiple sequence alignment showed that the 10 proteins were clustered into two distinct groups representing Gymnospermae and Angiospermae (Figure 2). GbF3′5′H1 had the closest relationships with *Taxus chinensis*, and the clustering analysis results were reliable. These two proteins were grouped with Gymnospermae, which is in accordance with the classification of these plant species. The other homologous proteins were relatively distantly related to GbF3′5′H1 and belonged to Angiospermae, but all the F3′5′H proteins of close relationships or the same family were clustered together. These relationships showed the evolutionary conservation and diversity of plant F3′5′Hs.

### 2.3. Expression Patterns of GbF3′5′H1

**Figure 3:**
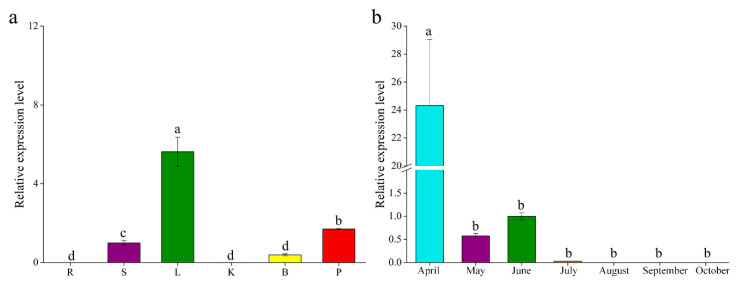
Gene expression patterns of *GbF3′5′H1* in *ginkgo* as determined by quantitative real-time PCR (qRT-PCR). (**a**) Expression patterns of *GbF3′5′H1* in various tissues: R = root, S = stem, L = leaf, K = kernel, B = bud, and P = petiole. The gene expression level in the stem was set to 1. *GbF3′5′H1* gene expression in *ginkgo* leaves at different times (from April to October). *GbF3′5′H1* gene expression level in June was set to 1. (**b**) Temporal expression pattern during April, May, June, July, August, September, and October. All the qRT-PCR data are shown as the mean ± standard deviation (error bar) of three biological replicates. Means with different letters are significantly different at *p* < 0.05, as determined by one-way ANOVA with Duncan’s multiple range tests.

To analyze the expression of *GbF3′5′H1*, we measured its transcript levels by quantitative real-time PCR (qRT-PCR) in different tissues and at different leaf stages in *ginkgo*. The expression analysis showed that *GbF3′5′H1* accumulated in the leaf (significantly more than in other tissues), followed by the petiole, and no expression was observed in the root and kernel (Figure 3a). There are seven stages in the development of *ginkgo* leaves from April to October (Figure 3b), and the accumulation of *GbF3′5′H1* transcripts was the highest in *ginkgo* leaves in April (more than 24 times higher than that in June and significantly higher than that in other periods), and the signal was notably weak in July (only 0.3 times its expression in June). There was no expression in the leaves from August to October.

### 2.4. Heterologous Overexpression of GbF3′5′H1 in Populus

**Figure 4:**
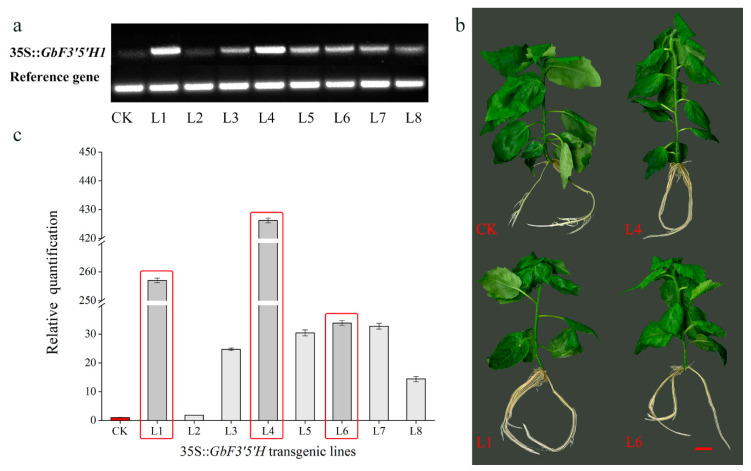
The expression levels of *GbF3′5′H1* in the eight transgenic poplar lines and nontransgenic (CK) *Populus* determined using semiquantitative qPCR (sqPCR) (**a**) and qRT-PCR (**b**). (**c**) Growth phenotypes of the different CK and transgenic seedlings at 45 days. Bar scale = 1.0 cm.

To investigate the function of *GbF3′5′H1* in vivo, multiple 35S:*GbF3′5′H1 Populus* transgenic lines were obtained and then validated by PCR. Eight independent transgenic lines were used to detect the expression level of *GbF3′5′H1* via semiquantitative qPCR (sqPCR) and qRT-PCR (Figure 4a,c). The results indicated that *GbF3′5′H1* was successfully expressed in all eight transgenic lines, and its expression levels were similar in the sqPCR and qRT-PCR analyses. The qRT-PCR results showed that the expression level of *GbF3′5′H1* in transgenic line 4 was the highest (the relative expression level was 426 times that of the control), followed by those of lines 1 and 6. The expression levels of all eight transgenic lines were higher than those of the CK poplars. After verifying the transgenic plants, we selected three transgenic overexpression lines (L1, L4, and L6) for subsequent experiments (Figure 4b). Moreover, we randomly selected six transgenic overexpression lines to observe their growth performance phenotypes at 45 days. The results showed no significant differences in the number of adventitious roots developed, maximum length of adventitious roots, and plant height compared with those of CK plants (Table S1). Hence, we concluded that there was very little difference in the growth phenotypes of the CK and transgenic plants at 45 days (Figure 4b).

### 2.5. Comparison of Flavonoid-Related Metabolites in CK and Transgenic Populus

Nontargeted gas chromatography-mass spectrometry (GC-MS) analysis showed that a total of 199 metabolites were identified (Supplementary Data 2). Among them, 45 significantly different metabolites were found between the CK and transgenic groups. The contents of 17 significantly different metabolites were higher in transgenic plants than that in CK plants. These 45 significantly different metabolites were mainly divided into the following nine super classes: organooxygen compounds (5); organic oxygen compounds (6); organic acids and derivatives (8); phenylpropanoids and polyketides (3); lipids and lipid-like molecules (3); nucleosides, nucleotides, and analogs (2); benzenoids (2); a homogeneous nonmetal compound (1); and an organoheterocyclic compound (1). In addition, the remaining 14 significantly different metabolites were unclassified. Four metabolites were closely related to flavonoid biosynthesis (Table 1). The contents of 4′,5-dihydroxy-7-glucosyloxyflavanone, epicatechin, and gallocatechin were significantly higher in the transgenic plants than in the CK plants by approximately 3.0, 2.5, and 2.3 times, respectively (Table 1). In total, three flavonoid-related metabolites showed significantly higher contents in the transgenic seedlings than in the CK.

## 3. Discussion

Flavonoids are among the most prevalent and biologically significant classes of secondary metabolites in *ginkgo* leaves [18,29]. In this study, we detected a differentially expressed *F3′5′H* gene known to be homologous to enzymes involved in the flavonoid synthesis pathway through transcriptome sequencing. A better understanding of the underlying molecular mechanism of flavonoid biosynthesis and its regulation could provide novel insights into improving the content of flavonoid-related metabolites. Thus, we cloned and characterized the *GbF3′5′H1* gene and assessed its expression in different tissues and at different leaf development stages. Moreover, we heterologously overexpressed *GbF3′5′H1* in *Populus* to investigate its function and then identified four metabolites closely related to flavonoid biosynthesis, including three metabolites with contents that were significantly higher in transgenic plants than in CK plants. These results confirmed that the *GbF3′5′H1* gene functions in the biosynthesis of flavonoid-related metabolites, especially flavan-3-ols.

### 3.1. Sequencing and Phylogenetic Analysis

In this study, a 1959-bp full-length cDNA of the *GbF3′5′H1* gene, encoding a 509 amino acid protein, was isolated from *ginkgo*. Our multiple alignment showed that the deduced *GbF3′5′H1* sequence exhibited high homology to the sequences of other plant *F3′5′H* proteins (Figure 1b). *F3′5′H* encodes a hydroxylase in the CYP family [30]. In this study, we found that the *GbF3′5′H1* gene contains several important structures, such as the C-terminal HBR, CYP motif, I helical region, and differential N-terminal amino acid sequence. This finding is consistent with other conserved sequences of *F3′5′H* proteins reported previously [3,28,31,32]. Among them, the C-terminal HBR “FGAGRRICAG” is a necessary sequence of CYP enzymes that is highly conserved among different species. This sequence is regulated by cysteine (Cys); this residue is at the center, and the amino acids to its left and right form specific structures around the Cys [33]. The CYP motif “PPGP” is also highly conserved in different species and serves as a hinge between the globular part of the protein and the membrane anchor [34,35]. The “AGTDT” of the I helical region is also highly conserved, and it is believed that this motif can promote the formation of oxygen molecules, acts as the binding region for proton transfer, and affects the selection and binding of substrates [36,37]. In addition, the differences in the amino acid sequences of *F3′5′Hs* in different species mainly lies in approximately 30 amino acids at the N terminus; this region is mainly hydrophobic. This sequence is the signal sequence indicating the membrane insertion point (known as a termination transfer signal), which mainly contains hydrophobic amino acid residues that can be anchored on the membrane [38], and this sequence is the main difference among the *F3′5′H* genes from different species. These facts indicate that *GbF3′5′H1* belongs to the CYP family and is highly homologous to *F3′5′H* sequences from other species.

Through gene structural analysis, we found that *GbF3′5′H1* had two exons and one intron (Figure 1a). Previous studies have shown that exons directly encode proteins and that introns affect RNA synthesis during transcription [39,40]. In addition, an interaction occurs between introns and the corresponding coding sequences after shearing, which plays an important role in the regulation of mRNA transport and gene expression [41,42,43]. Hence, the evolutionary and functional roles of the *GbF3′5′H1* intron remain to be studied. Moreover, *F3′5′H* protein sequences have been reported in divergent species across the plant kingdom [31,32,35,44]. In the present study, the phylogenetic tree indicated that *GbF3′5′H1* has a distinct and ancient relationship with the *F3′5′Hs* of other species in Gymnospermae, which is in accordance with the classifications of the plant species [45,46]. These results showed that the *F3′5′H1* protein in *ginkgo* is highly conserved and may have similar functions to those of the *F3′5′H* proteins in *Taxus chinensis*. These relationships reflect the evolutionary conservation and diversity of plant *F3′5′Hs*.

### 3.2. Differential Expression Patterns

*Ginkgo* is a widely used medicinal plant with high utilization value due to its flavonoids [20,47,48]. A pharmacological study by Ahlemeyer et al. (2003) indicated that the therapeutic use of *ginkgo* extract is beneficial for Alzheimer’s disease [20]. Moreover, *ginkgo* flavonoids was shown to treat cardiovascular diseases through diverse mechanisms [48]. Therefore, the differential expression patterns of the *GbF3′5′H1* gene, which is involved in the flavonoid biosynthetic pathway, could aid in further assessing the possible functions of flavonoids. Our expression analysis indicated that *GbF3′5′H1* accumulated in the leaf at a level significantly higher than that in other tissues (except for the aging leaves after September) (Figure 3a), implying that this gene might play important roles in the leaf. This finding is consistent with the higher content of flavonoids in *ginkgo* leaves than in other tissues, and the leaf of *ginkgo* is the most commonly used part of the plant, with the greatest medicinal value [18,49]. Previous studies indicated that the mRNA transcripts of *F3′5′H* accumulated in the petals of *Petunia hybrida* and *Pericallis* × *hybrida* [28,31]. This finding is different from the results of this study; this difference may be because *ginkgo* is a woody plant without petals, while the plants with high expression in the petals are herbaceous plants, mostly with ornamental value, and the petals are their most important ornamental parts. In addition, additional studies of *GbF3′5′H1* expression during leaf development showed that its expression was highest in April (Figure 3b). The differential expression of this gene during leaf development might be one of the key mechanisms underlying the production of flavonoids. Some studies have shown that the flavonoid content of *ginkgo* leaves was relatively high in May [18,50]. Thus, there may be a delay between the expression of this gene and the synthesis of flavonoids. The high expression of this gene in April and the accumulation and synthesis of flavonoids in *ginkgo* could gradually lead to a higher flavonoid content in May.

### 3.3. Functional Analysis

At present, research on genes involved in flavonoid synthesis in *ginkgo* is limited to in vitro enzyme activity analysis, gene expression analysis, and flavonoid content collaborative analysis [7,10,11,12], and no experiment has suggested the endogenous functions of these compounds in woody plants. Flavonoids are secondary metabolites unique to plants that have antioxidation, antitumor, and blood circulation-promoting effects [29,51]. Since no genetic transformation and regeneration system is available for *ginkgo*, this study investigated the biological function of the *GbF3′5′H1* gene by genetically transforming poplar, a woody model plant. After this gene was overexpressed in *Populus*, a nontargeted GC-MS analysis showed four metabolites closely related to flavonoid biosynthesis (Table 1), and the overexpression of the *GbF3′5′H1* gene in *Populus* promoted the production of three metabolites: 4′,5-dihydroxy-7-glucosyloxyflavanone, epicatechin, and gallocatechin. A functional analysis of *F3′5H* from *Camellia sinensis* by Wang et al., (2014) showed that *F3′5H* plays a critical role in the accumulation of catechins [52]. Although the results of this study are different from those of Wang et al., (2014), catechin, epicatechin, and epigallocatechin are flavan-3-ols. Taken together, these results showed that the *F3′5H* gene has a wide range of flavonoid substrate activities and suggested that the heterologous overexpression of *GbF3′5′H1* can produce a large amount of flavan-3-ol content.

Boase et al. [53] obtained the *F3′5′H* gene of cyclamen through rapid amplification of cDNA ends (RACE) cloning technology and changed the color of cyclamen petals by means of antisense suppression. Such reverse genetic methods will be helpful to further study the function of *GbF3′5′H1* and should be pursued in the future. The regulation of flavonoid metabolism is a complex process. The overexpression of one *GbF3′5′H1* gene was not expected to alter all of the wide variety of flavonoid compounds, but notably, three metabolites related to flavonoids were significantly increased in this study. *F3′5′H* competes with flavanone 3-hydroxylase, flavonoid 3′-hydroxylase, and flavonol synthase, and the competition among these enzymes affects the balance among the final products [44,54]. Therefore, we intend to further study the influences of other key enzyme-coding genes on flavonoid-related metabolites.

## 4. Materials and Methods

### 4.1. Plant Materials and Growth Conditions

*Ginkgo* trees grown at Nanjing Forestry University (118°81′’E, 32°08′’N, Jiangsu Province) were sampled. Leaves were collected at different developmental stages once per month from April to October to investigate gene expression patterns. After collection, the plant materials were rapidly frozen in liquid nitrogen and placed in an ultralow temperature freezer at −80 °C until use.

Tissue culture seedlings of the hybrid poplar, *Populus davidiana* × *Populus bolleana*, were grown at a temperature of 25 °C (day) and 18 °C (night) under a 16-h light and 8-h dark photoperiod. The cultured plantlets were cultivated on Murashige and Skoog medium (pH = 5.8) supplemented with 0.3% (*w*/*v*) Gelrite and 3.0% (*w*/*v*) sucrose.

### 4.2. Cloning of the GbF3′5′H1 Gene

Total RNA was extracted from *ginkgo* leaves using the RNAprep Pure Plant kit (Tiangen, Beijing, China). Specific primers were designed based on *ginkgo* transcriptome data [18] that included functional annotation of the *F3′5′H* protein. The full-length cDNA sequence of *GbF3′5′H1* was cloned using rapid amplification of cDNA ends (RACE) technology. Nested primers were designed to amplify full-length cDNA via the SMARTer RACE 5′/3′ Kit (Clontech, Japan) per the manufacturer’s manual. These primers (*GbF3′5′H1*_5′OUTER, *GbF3′5′H1*_3′OUTER, *GbF3′5′H1*_5′INNER, and *GbF3′5′H1*_3′INNER) were designed using Oligo 6.0 software (Table S2). Then, PCR products were separated by 1% agarose gel extraction and transformed into *Escherichia coli* competent cells using pMD19-T vector insertion (Takara, Japan). The colonies were checked using PCR, the positive colonies were selected, and Sanger sequencing was performed. The full-length cDNA sequencing of *GbF3′5′H1* was obtained by splicing the 5′ and 3′ RACE sequences, and NCBI ORF Finder was used to predict the open reading frame (ORF). The *GbF3′5′H1* ORF was amplified using the following PCR program: 95 °C for 3 min, 33 cycles of 95 °C for 30 s, 55 °C for 40 s, and 72 °C for 90 s, and a final extension at 72 °C for 10 min. Then, the target fragment was ligated into the pMD19-T vector and transformed into *Escherichia coli* TOP 10 cells. Positive clones were identified and sent for Sanger sequencing. In addition, the genomic DNA of *ginkgo* leaves was extracted using a Plant Genomic DNA Kit (cetyltrimethylammonium bromide (CTAB)) (Zoman, Beijing, China) to analyze the structural characteristics of the *GbF3′5′H1* gene.

### 4.3. Bioinformatics Analysis

DNA and protein sequence analyses were conducted using BioEdit. The structure of the *GbF3′5′H1* gene was visualized using the Gene Structure Display Server (http://gsds.cbi.pku.edu.cn/). The physical and chemical properties were predicted using ExPASy ProtParam (http://web.expasy.org/protparam/). The secondary structure was predicted by the online program self-optimized prediction method with alignment (SOPMA) (https://npsa-prabi.ibcp.fr/cgi-bin/npsa_automat.pl?page=npsa_sopma.html). Homologous sequences were obtained by searching for homologous alignments using the online Basic Local Alignment Search tool (BLAST) (https://blast.ncbi.nlm.nih.gov/). The deduced amino acid sequences and other downloaded protein sequences homologous to *F3′5′H* were used for multiple alignments using DNAMAN software. In addition, a phylogenetic tree was constructed using MEGA 7.0 software [55] by the maximum likelihood method based on a Poisson correction model [56] with 1000 bootstrap replications. The analysis included twelve amino acid sequences.

### 4.4. Quantitative Real-Time PCR (qRT-PCR) Analysis

To test the transcript expression levels of the *GbF3′5′H1* gene, qRT-PCR was carried out. Total RNA was extracted using an RNAprep Pure Plant kit (Tiangen, Beijing, China), which was then reverse-transcribed using a PrimeScript RT Master Mix (Takara, Dalian, China) to synthesize first-strand cDNA according to the manufacturer’s instructions. The cDNA was diluted 5 times as a template. Primers for qRT-PCR amplification of *GbF3′5′H1* (named *GbF3′5′H1*_qPCR in Table S2) and the internal reference gene (named the *ginkgo* reference gene in Table S2) were designed. A qRT-PCR analysis was performed using a FastStart Universal SYBR Green Master with 6-Carboxyl-X-Rhodamine (ROX) for the RT-PCR kit (Roche, Indianapolis, IN, USA) in accordance with the manufacturer’s instructions on the Applied Biosystems (ABI) ViiA 7 Real-time PCR platform. The reaction volume was 10 μL, and the PCR program was as follows: 95 °C for 2 min, 40 cycles at 95 °C for 15 s, and 95 °C for 1 min. Relative expression levels were calculated by the 2^−ΔΔCt^ method [57]. The glyceraldehyde-3-phosphate dehydrogenase gene (forward primer (5′-3′): GGTGCCAAAAAGGTGGTCAT and reverse primer (5′-3′): CAACAACGAACATGGGAGCAT) was used as a *ginkgo* reference gene. Elongation factor 1 alpha (forward primer (5′-3′): GGCAAGGAGAAGGTACACAT and reverse primer (5′-3′): CAATCACACGCTTGTCAATA) was used as a *Populus* reference gene. All data are expressed as the mean ± standard deviation. The data were analyzed using Duncan’s multiple range test in SPSS 22.0 software (SPSS Inc., Chicago, IL, USA). A *p*-value < 0.05 was considered statistically significant.

### 4.5. Transformation of Populus and Detection of Transgenic Populus Lines

The ORF of *GbF3′5′H1* was cloned into the entry vector pCR8/GW/TOPO (Invitrogen, USA). After verification by sequencing (primer named BP detection in Table S2), the fragment inserted in the entry vector was transferred to the destination vector pBI121 with a C-terminal HA-tag by an LR reaction (Table S2). The resulting vector (35S:*GbF3′5′H1*) was a high-copy vector with overexpression elements (35S promoter from the cauliflower mosaic virus (CaMV)). The constructed vector, 35S:*GbF3′5′H1*, was transformed into *Agrobacterium tumefaciens* strain EHA105 for the *Populus davidiana × Populus bolleana* transformation based on a previous method [58]. After screening using kanamycin resistance, CK and putatively transformed *Populus* lines were validated by semiquantitative qPCR (sqPCR) and qRT-PCR, as described above (named *GbF3′5′H1*_qPCR in Table S2).

### 4.6. Nontargeted Metabolic Assay

To determine whether the overexpression of *GbF3′5′H1* affected the synthesis of flavonoid-related metabolites in the transgenic plants, we used nontargeted metabolic analysis to detect differentially expressed metabolites and to determine the concentrations of metabolites in transgenic *Populus* leaves.

#### 4.6.1. Sample Preparation and GC-MS Processing

The leaves of three different CK *Populus* lines and three different transgenic *Populus* lines (L1, L4, and L6) were collected from plants grown under the same conditions. Notably, three transgenic clones of each transgenic line were collected by a mixed sampling method. In the Supplementary Data 2, GbF3′5′H1-1 represents the transgenic *Populus* line 4, GbF3′5′H1-2 represents the transgenic *Populus* line 1, and GbF3′5′H1-3 represents the transgenic *Populus* line 6. Each plant sample was accurately weighed to 60 mg and put into a 1.5-mL centrifuge tube, and 40 μL of internal standard (L-2- chlorophenylalanine, 0.3 mg/mL) was added. Two small steel balls and 360 μL of cold methanol were successively added and placed in the refrigerator at −80 °C for 2 min. Then, the balls were placed in a grinding machine and ground at 60 Hz for 2 min, and ultrasonic extraction was performed in an ice water bath for 30 min. Then, 200 μL of chloroform was added and vortexed (2 min), and 400 μL of water was added and vortexed (2 min). The samples were ultrasonically extracted in an ice water bath for 30 min and then incubated at −20 °C for 30 min. These samples were centrifuged at 12,000 rpm and 4 °C (10 min). The quality control (QC) sample was prepared by mixing aliquots of all samples to form a pooled sample. The sample was dried with a centrifugal concentrator, and 80 μL of 15-mg/mL methoxylamine hydrochloride in pyridine was subsequently added. The resultant mixture was vortexed vigorously for 2 min and incubated for 90 min at 37 °C. A total of 80 μL of N,O-bis (trimethylsilyl) trifluoroacetamide (BATFA) (with 1% trimethylchlorosilane (TMCS)) and 20 μL of *n*-hexane was added to the mixture, which was vortexed vigorously (2 min) and then derivatized at 70 °C (60 min).

All samples were placed at ambient temperature for 40 min before GC-MS analysis. The derivative samples were analyzed on an Agilent 7890B gas chromatography system with an Agilent 5977A MSD system (Agilent Technologies Inc., CA, USA). The subsequent GC-MS processing and analysis referred to the Chen et al. [59] study.

#### 4.6.2. Data Preprocessing and Statistical Analysis

ChemStation (version E.02.02.1431, Agilent, USA) software was used to convert the raw data (.D format) to. CDF format, and then the. CDF data were imported into ChromaTOF software (version 4.34, LECO, St Joseph, MI, USA) for data processing. The metabolites were annotated with the Fiehn or national institute of standards and technology (NIST) database. After alignment with the Statistic Compare component, raw data processing and statistical analysis were performed based on research by Ning et al. [60] and Xiong et al. [61], respectively.

#### 4.6.3. Selection of Differentially Expressed Metabolites

Differentially expressed metabolites were selected based on the combination of a statistically significant threshold of variable influence on projection (VIP) values obtained from the orthogonal partial least squares discriminant analysis (OPLS-DA) model and *p*-values from a two-tailed Student’s *t*-test on the normalized peak areas from different groups. Metabolites with VIP values larger than 1.0 and *p*-values less than 0.05 were considered differentially expressed metabolites.

## 5. Conclusions

In this study, we isolated and characterized the full-length 2051-bp *GbF3′5′H1* gene (full-length cDNA sequence was 1959-bp), which encodes a protein of 509 amino acids, from *ginkgo*. We found that the expression of *GbF3′5′H1* was the highest in the leaf and during April. Furthermore, nontargeted GC-MS analysis showed that the overexpression of *GbF3′5′H1* can increase the contents of the flavonoid-related metabolites 4′,5-dihydroxy-7-glucosyloxyflavanone, epicatechin, and gallocatechin in transgenic *Populus*; these results help to reveal the role of *GbF3′5′H1* in plant metabolism. Our findings contribute to a better understanding of the underlying molecular mechanism of flavonoid biosynthesis and its regulation and help to provide a scientific basis to improve the accumulation of flavonoids in plants.

## Figures and Tables

**Table 1:** The contents of flavonoid-related metabolites in *Populus*.

	4′,5-dihydroxy-7-glucosyloxyflavanone	Epicatechin	Epigallocatechin	Gallocatechin
Non-transgenic *Populus*	0.26 ± 0.12	0.02 ± 0.01	0.03 ± 0.00	0.07 ± 0.02
Transgenic *Populus*	0.77 ± 0.12 **	0.05 ± 0.01 *	0.04 ± 0.01	0.16 ± 0.01 **

Note: One-way analysis of variance with a post-hoc Duncan’s test with probabilities of *p* < 0.05 (*) and *p* < 0.01 (**).

## Supplementary Materials

The following are available online. Table S1: Statistics of adventitious root development and plant height in nontransgenic and transgenic *Populus* (45 d). Table S2: Primers for *GbF3′5′H1* gene cloning, vector construction, and expression analysis. Supplementary Data 1: The full-length cDNA and amino acid sequences of GbF3′5′H1. Supplementary Data 2: All metabolites were identified by nontargeted GC-MS analysis.
