# Supplementary material for: Overexpression of GbF3′5′H1 Provides a Potential to Improve the Content of Epicatechin and Gallocatechin
Source: Molecules. 2020 Oct 20;25(20):4836. doi: 10.3390/molecules25204836 (PMC7594021; doi:10.3390/molecules25204836)
Supplement: Supplementary file 1 [file molecules-25-04836-s001.zip › Supplementary Data/Supplementary Data 1.docx]

Supplementary Data 1

The full-length cDNA of GbF3'5'H1

aatatagcagcgagcaattgcagttgcagcgataacagcataATGGATCCGGAGACATTGAGAGAATTGCTGGTATGGGGGATTACATGGGCGGTATTGTACGTTGGGTTTCGTTATGTATTAAATTCGAGGAAGAAGAGAAAGTTGCCGCCAGGACCATCGGGATGGCCTTTGGTGGGTAGCCTGCCGTTGCTGGGACCGATGCCGCACGTAACGTTGTATAATCTGGCCAAGAAGCATGGGCCGATTCTCTATCTGAAGTTGGGTACATCGGCAATGGTGGTGGCTTCATCGCCTGAGACGGCCAAGGCGTTCTTGAAGACGTTGGACCTCAATTTCTCTAACAGACCGGGTAATGCCGGCGCAACCTATCTGGCCTATGATTCCCAAGACATGGTGTGGGCGCCCTACGGCCCTCGCTGGAAGATGCTACGCAAAGTGTGCAATCTCCACCTCTTGGGCGGGAAAGCGTTGGACGACTGGCAGCCGGTTCGAGAGGCGGAGATGGGTCACATGCTCCGTCTCATTCTCCAACACAGTTCTCGCCGCTCCAACCCGGTCGTTAATATACCCGAGATGCTCAACCTCTCCATGGCCAACATGCTTGGCCAGATCATTCTCAGCAAGCGCGTCTTCGCCACAGAGGGCGCCGAAGCCAACGAGTTCAAAGATATGGTGGTGGAGCTCATGACCTCCGCTGGCCTCTTCAACATCGGCGACTTCATTCCCTCCCTGGCCTGGATGGATCTCCAGGGTATCCAGCGGAACATGAAGAAGCTCCACAAGCGCTTCGATGCATTACTTACCCGCATGATCCAAGAGCATCAATCCTCTTCCCACCTAAGGCGTTCTCAAGACTTTCTCGACATCATCATGTCTCATCGAGAAAATGCCGACGGCGATGGCGGCCGCCTCACCGATGTCCACATCAAGAGTCTCCTACTGAATTTATTCACCGCTGGAACCGACACTTCGAGCAGCATCATAGAATGGGCGGTTGCGGAGCTGATACACAACCCAGAGATAGCCAAGAGAGCTCAAAGAGAAATGGACACAGTCATCGGACGCGAGAGAAAACTGAAGGAGTCGGACATAGCCAATCTGCCATACTTGGTGGCCATCTGCAAGGAGACGTTTCGGAAGCATCCTTCCACTCCGCTCAGTCTTCCTCGAGTGGCCGACCAAGACTGTCTGGTGGACGGATACTTCATCCCCAAGGACACCAAGCTCATGGTCAATGTGTGGGGCATCGGCAGAGACCCGGACCTGTGGGAGAAGCCATTGGAGTTCAATCCTGACAGGTTTCTGACGCCCAAAGGCTCCAAGATCGATCCCCGCGGAAACGATTTCGAGCTCATTCCCTTCGGTGCAGGGAGGCGCATCTGTGCAGGCACTCGCATGGGTATCAAATTGGTGGAGTACATCTTGGGCTCCCTAATCCATTCCTTCAATTGGGATCTTCCTCCAAACCAGAAGCAGCTCAACATGGATGAAGCTTTCGGCCTCGCCCTCCAGAAGGCTGTTCCTTTCGTCGCCACCCCTTCTCCTCGTCTTGCTCTCCATGTTTACTGAatcaaatttttattagtaagaccacatggaatatttagacattaaagggaaccgctttttctttggtggtttaggggatacgagggcctatttggtgtttaatgtgttgtagatgtattatcctgtttagagtttgagaatccagcggcatttagatcgaacgcacttgatttagtactcgctttataaaataagttttccttggattgttagctagattagattttgactctctatgcacttgtatgtgtaagggccatttcaactccattatggtaagttgaaatataaatatttgccacttacatgtaagggaatttattgagcacaagtcccaattaatttgactaaatcatttgccaaaaaaaaaaaaaaaaaaaaaaaaaa

The amino acid sequences of GbF3'5'H1

MDPETLRELLVWGITWAVLYVGFRYVLNSRKKRKLPPGPSGWPLVGSLPLLGPMPHVTLYNLAKKHGPILYLKLGTSAMVVASSPETAKAFLKTLDLNFSNRPGNAGATYLAYDSQDMVWAPYGPRWKMLRKVCNLHLLGGKALDDWQPVREAEMGHMLRLILQHSSRRSNPVVNIPEMLNLSMANMLGQIILSKRVFATEGAEANEFKDMVVELMTSAGLFNIGDFIPSLAWMDLQGIQRNMKKLHKRFDALLTRMIQEHQSSSHLRRSQDFLDIIMSHRENADGDGGRLTDVHIKSLLLNLFTAGTDTSSSIIEWAVAELIHNPEIAKRAQREMDTVIGRERKLKESDIANLPYLVAICKETFRKHPSTPLSLPRVADQDCLVDGYFIPKDTKLMVNVWGIGRDPDLWEKPLEFNPDRFLTPKGSKIDPRGNDFELIPFGAGRRICAGTRMGIKLVEYILGSLIHSFNWDLPPNQKQLNMDEAFGLALQKAVPFVATPSPRLALHVY
