# Supplementary material for: Overexpression of GbF3′5′H1 Provides a Potential to Improve the Content of Epicatechin and Gallocatechin
Source: Molecules. 2020 Oct 20;25(20):4836. doi: 10.3390/molecules25204836 (PMC7594021; doi:10.3390/molecules25204836)
Supplement: Supplementary file 1 [file molecules-25-04836-s001.zip › Supplementary Data/Table S1.2.docx]

Supplementary Data

**Table S1.** Statistics of adventitious root development and plant height in non-transgenic and transgenic *Populus* (45 d).

| Name | Number of adventitious roots | Maximum length of adventitious root | Plant height |
| --- | --- | --- | --- |
| Wild type *Populus* | 5.00±0.63 | 6.30±0.62 | 7.33±0.48 |
| Transgenic *Populus* | 4.83±1.47 | 6.37±0.74 | 7.27±1.09 |

**Table S2.** Primers for *GbF3'5'H1* gene cloning, vector construction and expression analysis.

| Primer_ID | Forward PCR primer (5'-3') | Reverse PCR primer (5'-3') |
| --- | --- | --- |
| *GbF3'5'H1*_5'OUTER | CTAATACGACTCACTATAGGGCAAGCAGTGGTATCAACGCAGAGT | GAGGAAGACTGAGCGGAGTGGA |
| *GbF3'5'H1*_5'INNER | CTAATACGACTCACTATAGGGC | TTGGCTATGTCCGACTCCTT |
| *GbF3'5'H1*_3'OUTER | CCTCGCTGGAAGATGCTACGCA | ACTCTGCGTTGATACCACTGCTTGCCCTATAGTGAGTCGTATTAG |
| *GbF3'5'H1*_3'INNER | TTGGGCGGGAAAGCGTTGG | GCCCTATAGTGAGTCGTATTAG |
| *GbF3'5'H1*_qPCR | ACACAACCCAGAGATAGCCAA | TGAGCGGAGTGGAAGGATG |
| BP detection | ATGAAGAGCTTTGCTAGT | TAATACGACTCACTATAGGG |
| LR detection | CGCACAATCCCACTATCCTT | CTAGGATAGCTTCCAAGCTTTACCA |
| *Ginkgo* reference gene | GGTGCCAAAAAGGTGGTCAT | CAACAACGAACATGGGAGCAT |
| *Populus* reference gene | GGCAAGGAGAAGGTACACAT | CAATCACACGCTTGTCAATA |
